# Validation of the Childhood Family Mealtime Questionnaire in Mexican Adolescents with Obesity and Their Caregivers

**DOI:** 10.3390/nu15234937

**Published:** 2023-11-28

**Authors:** Melissa Silva-Uribe, Fernanda Máynez-López, Edgar Denova-Gutiérrez, Brenda Muñoz-Guerrero, Isabel Omaña-Guzmán, Sarah E. Messiah, Arturo Ruíz-Arroyo, Emilia Lozano-González, Eréndira Villanueva-Ortega, Paloma Muñoz-Aguirre, Nayely Garibay-Nieto

**Affiliations:** 1 Pediatric Obesity Clinic and Wellness Unit, Hospital General de México “Dr. Eduardo Liceaga”, Mexico City 06720, Mexico; mel.silvaa1193@gmail.com (M.S.-U.); mfernanda.maynez.lopez@gmail.com (F.M.-L.); brend_rocc@hotmail.com (B.M.-G.); nutricion.isa@gmail.com (I.O.-G.); ruizaj22@gmail.com (A.R.-A.); emilia.lozano.el@gmail.com (E.L.-G.); villorte@yahoo.com (E.V.-O.); 2 Centro de Investigación en Nutrición y Salud, Instituto Nacional de Salud Pública, Cuernavaca 62100, Mexico; edgar.denova@insp.mx; 3 Center for Pediatric Population Health, University of Texas Health Science Center at Houston School of Public Health, Dallas, TX 75207, USA; sarah.e.messiah@uth.tmc.edu; 4 Consejo Nacional de Humanidades, Ciencias y Tecnologías (CONAHCYT), Mexico City 03940, Mexico; paloma.munoz@insp.mx; 5 Centro de Investigación en Salud Poblacional, Instituto Nacional de Salud Pública, Cuernavaca 62100, Mexico

**Keywords:** childhood family mealtime questionnaire, obesity, adolescents, validity

## Abstract

Background: Childhood obesity is a significant public health concern in Mexico, with far-reaching implications for the nation’s healthcare system and economy. In light of this challenge, our study sought to validate the Childhood Family Mealtime Questionnaire (CFMQ) in Mexican adolescents living with obesity and their primary caregivers. Methods: A sample of 56 adolescents ages 13 to 17 years and their primary caregivers from one pediatric obesity clinic participated in the study. We conducted a comprehensive assessment of the CFMQ’s consistency, reliability, and construct validity among all participants. Internal consistency was determined using Cronbach’s α, and the questionnaire’s reliability was assessed through test–retest and intraclass correlation coefficients. Construct validity was assessed through an exploratory factor analysis. Results: Our findings confirmed strong internal consistency and reliability for both adolescents and caregivers. Construct validity was established through exploratory factor analysis, refining the questionnaire while preserving its original seven dimensions. This validation of the CFMQ highlights its applicability in evaluating family mealtime experiences in this context, providing valuable insights into the dynamics that influence adolescent nutrition and health. Conclusion: The CFMQ proves to be a reliable tool for assessing family mealtime experiences in Mexican adolescents living with obesity and their caregivers who seek care at third-level public hospitals.

## 1. Introduction

Obesity, a complex, multifactorial, and preventable disease, along with overweight, afflicts approximately one-third of the global population [1]. According to data from the Mexican National Health and Nutrition Survey 2020–2022 (ENSANUT), the combined prevalence of overweight and obesity among Mexican adolescents aged 12 to 19 years was 41.1% [2]. These prevalence estimates impose a substantial economic burden on the country, with reports suggesting obesity accounted for an economic impact of 2.1 percent on the gross domestic product (GDP) in 2019, while it is projected to more than double to as much as 4.7 percent of GDP by 2060 if urgent measures are not instituted across all levels of society [3].

Family dynamics and functioning have been strongly identified as crucial risk factors linked to the development of obesity and as impediments to successful interventions [4,5,6]. In 2007, Barlow et al. [7] proposed that comprehensive strategies incorporating family-based interventions, as well as changes in weight-related behaviors rather than weight loss goals, can lead to sustained improvements in medical and psychosocial outcomes for children and adolescents living with obesity. Numerous studies have delved into how family behaviors associated with lifestyle, such as diet, physical activity, feeding practices, parental roles, health care support, and parenting style, may influence the weight of adolescents [4,5,8,9,10,11]. However, only a limited number of studies have explored the impact of broader family environmental factors, such as family functioning during mealtimes or the quality of the relationship between parents and adolescents, on the body composition and lifestyle of adolescents [6,12,13].

In clinical practice, the customary method for assessing family functioning is to rely on independent reports provided by adolescents and caregivers. Nevertheless, Lebron et al. [14] reported data showing disparities between the perceptions of adolescents and their caregivers regarding family functioning when using the Childhood Family Mealtime Questionnaire (CFMQ). This underscores the significance of evaluating both perspectives using the same tool. In Mexican clinical practice, there is a current absence of validated questionnaires tailored for assessing family mealtime experiences in children and adolescents, together with their primary caregivers. The integration of such validated tools within clinical settings stands to empower practitioners in devising and executing personalized treatments, effectively meeting the distinct needs of each individual and their family. This integration holds promise for fostering sustainable positive outcomes and advancing a more nuanced approach to healthcare interventions. Therefore, our objective is to validate the CFMQ in Mexican adolescents living with obesity and their caregivers. 

## 2. Materials and Methods

### 2.1. Study Design and Subjects 

This cross-sectional analysis is a component of an ongoing study titled “Association of Discrepancies in Family Functioning and Dietary Patterns in Adolescents Living with Obesity”. This project is being conducted at the Pediatric Obesity Clinic and Wellness Unit, a renowned center for pediatric obesity management located at the Hospital General de México, which falls under the jurisdiction of the Ministry of Health and provides services to the population without social security.

Study participants included adolescents aged 13 to 17 years, living with overweight or obesity, as defined by the Centers for Disease Control and Prevention standards [15], and their primary caregivers, who were attending the Pediatric Obesity Clinic and Wellness Unit for the first time. Participants who were unable to complete the questionnaire or had missed either of the two questionnaire administrations were excluded. Prior to their participation in the study, both adolescents and caregivers provided written informed assent and consent, respectively. The study was conducted in accordance with the guidelines outlined in the Declaration of Helsinki and received approval from the Research and Bioethics Committees at Hospital General de México “Dr. Eduardo Liceaga” (DI/21/303/05/20).

### 2.2. Power Calculation

We determined an estimated sample size of 40 adolescents and their corresponding caregivers to evaluate the significance of the Pearson linear correlation coefficient. This calculation was based on an effect size of 0.3, an alpha level of 0.05, and a statistical power of 0.80, as estimated by GPower 3.1 software. Factoring in a 30% allowance for potential loss to follow-up, it was concluded that the final sample should consist of at least 52 adolescents and their caregivers. Consequently, the final sample comprised 56 dyads of adolescents and caregivers.

### 2.3. Procedures

#### 2.3.1. Primary Outcome Measure

##### Childhood Family Mealtime Questionnaire (CFMQ)

Our main outcome measure was the complete 69-item version of the CFMQ, specifically designed to evaluate mealtime experiences related to eating disorders in women [16]. However, this tool has found utility in investigating various early disorders associated with early food experiences, which is particularly valuable given the connections between obesity and eating disorders [17,18,19]. Furthermore, it has received validation for use in adolescents living with obesity [20]. The CFMQ encompasses seven distinct constructs: (1) Mealtime Communication Based Stress, (2) Mealtime Structure, (3) Appearance Weight Control, (4) Parental Mealtime Control, (5) Emphasis on Mother’s Weight, (6) Present Parental Meal Influence, and (7) Traditional Family [16].

The CFMQ was administered to both adolescents and their caregivers (one caregiver per adolescent) on two occasions by a standardized interviewer. The initial administration took place upon enrollment, while the second occurred one month later. In all instances, the CFMQ was administered to adolescents at the clinic. Depending on their availability, the CFMQ was administered to caregivers at the clinic facilities or via email with instructions for digital completion. The time lapse between responses averaged 14 days, ranging from 4 days to 30 days.

##### CFMQ Validation

We conducted a comprehensive assessment of the CFMQ’s consistency, reliability, and construct validity in both adolescents and their primary caregivers. Internal consistency was determined using Cronbach’s α, and the questionnaire’s reliability was assessed through test–retest and intraclass correlation coefficients. Construct validity was scrutinized by employing an exploratory factor analysis, with a focus on the original seven dimensions of the CFMQ; thus, this analysis involved the examination of seven factors. Further elaboration on the statistical analyses undertaken is presented in the subsequent section.

##### Adolescent Measures

Sociodemographic Variables: At enrollment, a questionnaire was employed to collect sociodemographic data, including birthdate, educational level (no education, elementary school, middle school, and high school), and residential area (Mexico City, State of Mexico, metropolitan area, and other). The age in years was calculated by subtracting the participant’s self-reported birthdate from the survey completion date.

Anthropometric Measurements: Weight (in kilograms) was determined using a Body Composition Analyzer Jawon, IOI 353 (to 0.1 kg), while height (in centimeters) was measured with a mobile stadiometer SECA 217 (to 0.1 cm). Body mass index (BMI) was calculated by dividing the weight in kilograms by the square of the height in meters (kg/m^2^), and BMI Z-scores were calculated using the World Health Organization (WHO) Anthro Plus Software^®^ 1.0.4 [21,22]. Overweight was defined as BMI ≥85th percentile, and obesity was defined as BMI ≥ 95th percentile, both adjusted for age and sex, according to CDC 2000 criteria [15]. 

Clinical Measurements: A pediatrician assessed adolescents to determine their Tanner stage on a scale ranging from 1 (no development) to 5 (full development) for genital (males), breast (females), and pubic hair (both) development. Pubertal status was categorized as prepubertal (Tanner stage 1), early puberty (Tanner stages 2 or 3), or advanced puberty (Tanner stages 4 or 5) [23].

### 2.4. Caregiver Measures

Sociodemographic Variables: At enrollment, caregivers completed a questionnaire providing sociodemographic information, including age, marital status (single, married/consensual union, separated/divorced, widow), educational level (no education, less than high school, high school, some college, college), and residential area (Mexico City, State of Mexico, metropolitan area, and other).

Anthropometric Measurements: Weight in kilograms and height in centimeters were assessed using the same equipment utilized for adolescent participants (Body Composition Analyzer Jawon, IOI 353, and a mobile stadiometer SECA 217).

### 2.5. Statistical Analysis

Descriptive statistics including frequencies and percentages (for categorical variables) and the means and standard deviations (for continuous variables) of the sociodemographic and clinical characteristics within the study population were calculated.

To assess CFMQ internal consistency, Cronbach’s α and the correlation between items were calculated both for the entire scale and for each dimension. The interpretation of Cronbach’s α values was as follows: values below 0.70 indicated that individual items did not sufficiently contribute to the overall scale, while values exceeding 0.90 suggested redundancy [24]. 

Reliability was assessed through a test–retest, and intraclass correlation coefficients (ICCs) were computed, along with their 95% confidence intervals (CIs), based on a two-way mixed effect, absolute agreement, single rater/measurement model. ICCs were evaluated using the following cut-off points: low reliability (<0.5), moderate reliability (0.50–0.75), good reliability (0.75–0.90), and excellent reliability (>0.90) [24].

Construct validity was determined through exploratory factor analysis with varimax rotation. The adequacy of the sample was confirmed by considering the Kaiser–Mayer–Olkin (KMO) measure (with an appropriate value >0.5), and the use of factor analysis was supported by Bartlett’s test of sphericity (with a significant value of *p* < 0.05). The number of factors was determined based on eigenvalues greater than 1. Item-factor membership was identified by factor loadings higher than 0.20, indicating the degree of association of each item with each factor [25]. 

All statistical analyses were performed using STATA 17.0 for Windows, and a significance level of *p* < 0.05 was considered statistically significant.

## 3. Results

### 3.1. Characteristics of Adolescents and Caregivers

The final analytical sample included a total of 56 adolescents (55.4% female, mean age 14.8 years (±1.4)) and their primary caregivers. The majority (88.6%) of adolescents were categorized as having late puberty. Mean BMI Z-score was 2.32 (±0.57), with no significant difference between males and females (2.33 and 2.32, respectively). All caregivers were women, with an average age of 42.9 years (±6.4). Approximately 11% held a bachelor’s degree, and mean BMI was 31.5 kg/m^2^ (±5.3) (Table 1).

### 3.2. Validation

#### 3.2.1. Internal Consistency and Reliability

Table 2 displays the outcomes for internal consistency (Cronbach’s α) and reliability/test–retest (ICC and 95% CI) for each dimension of the questionnaire in adolescents. Overall, the CFMQ demonstrated strong internal consistency (Cronbach’s α = 0.84) and reliability (ICC = 0.86; 95% CI: 0.82, 0.90). When considering the questionnaire’s dimensions, mealtime structure exhibited the highest consistency and reliability (Cronbach’s α = 0.88 and ICC = 0.90; 95% CI: 0.85, 0.95). In contrast, the appearance weight control dimension had the lowest consistency (Cronbach’s α = 0.78) and reliability (ICC = 0.79; 95% CI: 0.74, 0.85).

For caregivers, the entire CFMQ also exhibited strong internal consistency (Cronbach’s α = 0.86) and reliability (ICC = 0.88; 95% CI: 0.84, 0.92). Similarly to what was observed in adolescents, the mealtime structure dimension showed the highest consistency and reliability (Cronbach’s α = 0.89 and ICC = 0.91; 95% CI: 0.87, 0.95), whereas the appearance weight control dimension displayed the lowest consistency (Cronbach’s α = 0.81) and reliability (ICC = 0.82; 95% CI: 0.79, 0.85) (Table 3).

#### 3.2.2. Construct Validity 

Construct validity was evaluated through exploratory factor analysis, with summarized results presented in Table 4 and Table 5. In the case of adolescents, a KMO measure of 0.89 and a significant result (*p* < 0.001) for the Bartlett sphericity test confirmed the adequacy of the sample. For caregivers, the KMO measure was 0.92, with a significant result (*p* < 0.001) for the Bartlett sphericity test, also affirming the sample’s adequacy. In both instances, the initial questionnaire, comprising 69 items, was reduced to 35 items, considering factor loadings greater than 0.20. These items were grouped into seven factors: I. Mealtime Communication Based Stress, II. Mealtime Structure, III. Appearance Weight Control, IV. Parental Mealtime Control, V. Emphasis on Mother’s Weight, VI. Present Parental Meal, and VII. Traditional Family. This seven-factor structure accounted for approximately 88% of the total variance, with all factors exhibiting eigenvalues greater than 1.

## 4. Discussion

The Childhood Family Mealtime Questionnaire (CFMQ) demonstrated commendable consistency, reliability, and construct validity among Mexican adolescents and their caregivers. This indicates that the CFMQ effectively measures the constructs it was designed for, and these properties remain stable over time. Consequently, the CFMQ was shown to be a valuable tool for use in the target study population.

The seven factors theoretically corresponding to the original seven domains that the CFMQ aims to assess in adolescents and their caregivers accounted for 88% of the variance. Based on this explained variance, in conjunction with the KMO and Bartlett tests and the theoretical underpinnings of each item, we can confidently conclude that the seven-factor model identified aligns well with our data. Furthermore, the analysis of internal consistency and reliability revealed that items within each domain exhibited strong correlations with their respective domains, and the instrument’s ability to measure these dimensions consistently withstood the test of time.

While originally designed to evaluate mealtime experiences related to eating disorders in women [16], our results suggest that the CFMQ can be effectively applied to Mexican adolescents with obesity. This aligns with existing evidence indicating shared risk behaviors between obesity and eating disorders, including body dissatisfaction, dieting, weight-control behaviors, and similar family environmental characteristics [26,27,28]. Moreover, it is known that obesity serves as a risk factor for the development of eating disorders [29]. 

The theoretical dimensions of the CFMQ are intricately linked to familial behaviors that may either heighten or mitigate the risk of unhealthy food habits, subsequently influencing the likelihood of obesity in children and adolescents. For example, the Mealtime Communication-Based Stress dimension assesses communication and stress management skills during shared family mealtimes. Positive experiences in such settings have been correlated with a reduced risk of obesity and eating disorders in previous studies [30].

The Appearance Weight Control and Emphasis on Mother’s Weight dimensions highlight family and individual behaviors associated with body dissatisfaction and an excessive concern about body weight. These factors have been identified as contributors to childhood obesity and eating disorders [29,31].

Compelling studies emphasize the significance of family mealtime habits, demonstrating that shared meals correlate with healthier dietary choices for all family members and a decreased risk of eating disorders. The frequency of shared family meals has been explored in relation to the nutritional health of children and adolescents [13]. Other research underscores the positive impact of reinforcing communication between parents and children during meals, along with providing positive parental food reinforcement [30], emphasizing the interconnectedness of childhood obesity and interpersonal dynamics during family meals. Moreover, studies have highlighted the increased probability of consuming unhealthy foods at various times of the day in the absence of structured feeding schedules [31]. Communication challenges within a family regarding eating habits may lead adolescents to prefer isolation during meals, prefer larger portions of either healthy or unhealthy foods, and spend additional hours in front of screens. These behaviors can negatively impact their mood, perpetuating unhealthy relationships between family members.

Considering the above, the CFMQ emerges as a valuable tool for assessing family mealtime experiences in adolescents living with obesity.

Nevertheless, several study limitations must be acknowledged. First, there was variability in response times between the test and retest, ranging from four days to one month. Despite this, we observed a strong level of reliability (ICC = 0.86; 95% CI: 0.82, 0.90). Additionally, we were unable to explore the correlations between CFMQ items and domains with related variables to bolster construct validity. Lastly, the study population exhibited specific socioeconomic and clinical characteristics, limiting the generalizability of our findings to other populations. Nonetheless, these results hold relevance for clinical settings with similar characteristics, such as third-level public clinics attended by adolescents living with obesity who lack access to the social security system.

On the positive side, our sample size provided sufficient statistical power (80%) for conducting the validation of the CFMQ, and the employed statistical methods were robust, thereby enhancing the reliability of our findings.

To the best of our knowledge, this is the first validation study of the CFMQ in Mexican adolescents and their caregivers. The validation of this tool in adolescents and their caregivers facilitates a comprehensive assessment of mealtime experiences, capturing both adolescent and caregiver perceptions and enabling the identification of response discrepancies. This, in turn, sheds light on the family dynamics that influence the nutrition and health status of adolescents. Recognizing the barriers and facilitators within family dynamics related to mealtimes is essential for designing and implementing interventions tailored to the individual needs and family context of each patient, as recommended by current clinical practice guidelines [7,9,32]. Psychosocial factors, notably family behaviors, play a pivotal role in both the development and persistence of obesity. The dimensions of the CFMQ provide health professionals with a valuable tool to identify family behaviors that hinder the establishment of healthy food habits and contribute to the prevalence of eating disorders among adolescents with obesity. Future research could focus on identifying specific communication discrepancies between adolescents and their family members through the CFMQ. This nuanced understanding could serve as a complementary approach, enhancing our ability to identify and address barriers that may impede their adherence to a healthy lifestyle intervention program.

## 5. Conclusions

In summary, this study shows that the Childhood Family Mealtime Questionnaire (CFMQ) is a reliable tool for assessing family mealtime experiences in Mexican adolescents living with obesity and their caregivers who seek care at third-level public hospitals. It is imperative to extend validation of the CFMQ to diverse clinical settings in Mexico to ensure its broader applicability.

The presence of a validated questionnaire for evaluating the family mealtime environment is of paramount importance in comprehending the factors associated with obesity at both the individual and household levels. This, in turn, empowers the enhancement of interventions aimed at preventing and treating childhood obesity.

## Figures and Tables

**Table 1:** Characteristics of adolescent and caregiver participants.

Adolescent Characteristics (*n* = 56)	*n* (%)
**Sex**	
Male	25 (44.6)
Female	31 (55.4)
**Pubertal maturity**	
Prepubertal (Tanner I)	1 (2.3)
Early puberty (Tanner II, III)	5 (9.1)
Late puberty (Tanner IV, V)	50 (88.6)
**Education level**	
No education	2 (2.6)
Elementary school	7 (13.2)
Middle school	25 (44.7)
High school	22 (39.5)
Age (years) mean (SD)	14.8 (1.4)
BMI (kg/m^2^) mean (SD)	29.5 (3.9)
BMI percentile mean (SD)	97.5 (2.9)
zBMI mean (SD)	2.3 (0.6)
**Caregiver characteristics (*n* = 56)**	***n* (%)**
**Sex**	
Female	100%
**Marital status**	
Single	13 (21.7)
Married/cohabitation	29 (52.2)
Separated/divorced	13 (23.9)
Widow	1 (2.2)
**Education level**	
No education	0 (0)
Less than high school	33 (58.7)
High school	16 (28.3)
Some college	1 (2.2)
College	6 (10.9)
**Residence**	
Mexico City	28 (50)
State of Mexico and metropolitan area	25 (45.7)
Other	3 (4.3)

BMI, body mass index; SD, standard deviation.

**Table 2:** Reliability and internal consistency of CFMQ in adolescents.

Questionnaire Dimension	Cronbach’s α	Inter-Item Correlations	Intraclass Correlation Coefficient (95% CI)
I. Mealtime Communication-Based Stress	0.84	0.81	0.86 (0.82, 0.90)
II. Mealtime Structure	0.88	0.85	0.90 (0.85, 0.95)
III. Appearance Weight Control	0.78	0.76	0.79 (0.74, 0.85)
IV. Parental Mealtime Control	0.79	0.71	0.81 (0.77, 0.85)
V. Emphasis on Mother’s Weight	0.80	0.79	0.81 (0.76, 0.87)
VI. Present Parental Meal	0.79	0.77	0.80 (0.73, 0.86)
VII. Traditional Family	0.85	0.83	0.88 (0.83, 0.93)

CI, confidence interval.

**Table 3:** Reliability and internal consistency of CFMQ in caregivers.

Questionnaire Dimension	Cronbach’s α	Inter-Item Correlations	Intraclass Correlation Coefficient (95% CI)
I. Mealtime Communication-Based Stress	0.85	0.83	0.87 (0.82, 0.92)
II. Mealtime Structure	0.89	0.82	0.90 (0.85, 0.95)
III. Appearance Weight Control	0.81	0.79	0.91 (0.87, 0.95)
IV. Parental Mealtime Control	0.84	0.79	0.83 (0.79, 0.87)
V. Emphasis on Mother’s Weight	0.83	0.80	0.84 (0.78, 0.89)
VI. Present Parental Meal	0.84	0.81	0.85 (0.78, 0.92)
VII. Traditional Family	0.87	0.84	0.88 (0.82, 0.92)

CI, confidence interval.

**Table 4:** Factorial analysis in construct validity of CFMQ in adolescents.

Questionnaire Dimension	Factor Loadings						
	I	II	III	IV	V	VI	VII
**I. Mealtime Communication-Based Stress**							
I like to eat with my family	0.73						
At mealtime, we can all share our points of view	0.72						
It is a relief when my father is not around for lunch	0.79						
I prefer to eat alone to avoid the stress of eating as a family	0.66						
During mealtime, I feel comfortable enough to share my views	0.69						
I feel that family meals are pleasant and a moment to share with the family	0.62						
I look forward to lunchtime with enthusiasm	0.45						
During mealtime, you yell	0.49						
I remember feeling nervous during mealtime	0.59						
During mealtime, my family talks	0.38						
In my family, mealtimes are spent in silence	0.44						
**II. Mealtime Structure**							
In my house, I have to clean my plate (for example, eat everything that is served to us)		0.67					
My family is aware of not wasting food		0.61					
My parents force me to eat foods that I do not like		0.80					
My parents care a lot about having good manners		0.32					
They tell me not to waste food during mealtime		0.53					
I have to eat what they serve me even if I do not like it		0.50					
Manners are discussed during mealtime		0.45					
**III. Appearance Weight Control**							
In my family, we comment on our own weight and that of others			0.72				
I care about my weight			0.54				
I am worried about my weight			0.49				
They motivate me to diet			0.61				
My family believes that beauty has a lot to do with weight			0.52				
There is a lot of talk/importance about physical appearance in my family			0.70				
**IV. Parental Mealtime Control**							
During mealtime, it is easy to see who’s in control of my family				0.78			
We eat foods that my father likes				0.62			
Food is part of family celebrations				0.50			
**V. Emphasis on Mother’s Weight**							
My mother diets					0.58		
My mother worries/worried about her weight/shape					0.85		
My father makes comments about my mother’s weight					0.79		
**VI. Present Parental Meal**							
My parents still influence what I eat while I am at home						0.64	
My parents still influence the way I eat while I am at home						0.32	
**VII. Traditional Family**							
The role of cook is one of my mother’s main roles							0.67
I consider my mother to be primarily a homemaker							0.47
My father’s schedule establishes mealtimes for my family							0.29

**Table 5:** Factorial analysis in construct validity of CFMQ in caregivers.

Questionnaire Dimension	Factor Loadings						
	I	II	III	IV	V	VI	VII
**I. Mealtime Communication-Based Stress**							
I like to eat with my family	0.70						
At mealtime, we can all share our points of view	0.78						
It is a relief when my father is not around for lunch	0.80						
I prefer to eat alone to avoid the stress of eating as a family	0.45						
During mealtime, I feel comfortable enough to share my views	0.69						
I feel that family meals are pleasant and a moment to share with the family	0.62						
I look forward to lunchtime with enthusiasm	0.35						
During mealtime, you yell	0.69						
I remember feeling nervous during mealtime	0.39						
During mealtime, my family talks	0.48						
In my family, mealtimes are spent in silence	0.54						
**II. Mealtime Structure**							
In my house, I have to clean my plate (for example, eat everything that is served to us)		0.68					
My family is aware of not wasting food		0.71					
My parents force me to eat foods that I do not like		0.70					
My parents care a lot about having good manners		0.62					
They tell me not to waste food during mealtime		0.33					
I have to eat what they serve me even if I do not like it		0.40					
Manners are discussed during mealtime		0.55					
**III. Appearance Weight Control**							
In my family, we comment on our own weight and that of others			0.49				
I care about my weight			0.65				
I am worried about my weight			0.72				
They motivate me to diet			0.41				
My family believes that beauty has a lot to do with weight			0.32				
There is a lot of talk/importance about physical appearance in my family			0.80				
**IV. Parental Mealtime Control**							
During mealtime, it is easy to see who’s in control of my family				0.70			
We eat foods that my father likes				0.82			
Food is part of family celebrations				0.40			
**V. Emphasis on Mother’s Weight**							
My mother diets					0.46		
My mother worries/worried about her weight/shape					0.74		
My father makes comments about my mother’s weight					0.65		
**VI. Present Parental Meal**							
My parents still influence what I eat while I am at home						0.68	
My parents still influence the way I eat while I am at home						0.46	
**VII. Traditional Family**							
The role of cook is one of my mother’s main roles							0.73
I consider my mother to be primarily a homemaker							0.64
My father’s schedule establishes mealtimes for my family							0.31

## Data Availability

The data presented in this study are available on request from the corresponding author.
